# Myogenic Differentiation and Immunomodulatory Properties of Rat Adipose-Derived Mesenchymal Stem/Stromal Cells

**DOI:** 10.3390/biology13020072

**Published:** 2024-01-25

**Authors:** Sai Koung Ngeun, Miki Shimizu, Masahiro Kaneda

**Affiliations:** 1 Laboratory of Veterinary Diagnostic Imaging, Faculty of Agriculture, Tokyo University of Agriculture and Technology, 3-5-8 Saiwai-cho, Fuchu, Tokyo 183-8509, Japan; s212892q@st.go.tuat.ac.jp; 2 Laboratory of Veterinary Anatomy, Faculty of Agriculture, Tokyo University of Agriculture and Technology, 3-5-8 Saiwai-cho, Fuchu, Tokyo 183-8509, Japan; kanedam@cc.tuat.ac.jp

**Keywords:** adipose tissues, gene expression, immunocytochemistry, mesenchymal stem/stromal cells, myogenesis

## Abstract

**Simple Summary:**

Muscular degeneration is a prevalent disease with a challenging prognosis. Mesenchymal stem/stromal cells (MSCs) show promise in treating muscular degeneration due to their myogenic differentiation potential and immunomodulatory capabilities. Adipose tissues have emerged as a promising source of MSCs as a result of their easy accessibility and superior quality compared to other sources. However, their characteristics and differentiation ability vary among the sources and species of origin and remain controversial. Understanding these features is crucial for effectively treating muscular diseases using adipose-derived MSCs (ADP MSCs). To deepen our comprehension, we used the rat model to investigate the myogenic differentiation potential and immunomodulatory characteristics of ADP MSCs isolated from periscapular fat. ADP MSCs expressed key genes involved in immunomodulation. They also differentiated into myogenic cells and expressed myogenic markers. These findings suggest the promising clinical potential of ADP MSCs for muscle regeneration.

**Abstract:**

The myogenic differentiation potential of MSCs is a key factor in their potential use as a cell source for muscle tissue repair and regeneration. Additionally, evaluating the immunomodulatory properties of MSCs is important to highlight their potential for regulating inflammation and supporting tissue regeneration. Given the limited literature on muscle differentiation potential and immunomodulatory properties, this study aims to characterize rat ADP MSCs for treating muscle disease. We isolated MSCs from adipose tissues around the periscapular region of the rats. We used a monoculture method for the myogenic differentiation and modified the myogenic induction medium by supplementing it with the growth factors FGF, HGF, and IGF. In rat ADP MSCs, expression of the MSC-specific marker, CD90, was 87.7%, while CD44 was 42.8%. For genes involved in immunomodulation, *IGF1* and *TGFB1* were highly expressed, while *IL6* was poorly expressed. In addition to their trilineage differentiation potential, ADP MSCs exhibited the capacity to differentiate into myogenic cell lines, as evidenced by changes in cell morphology, leading to elongated and aligned structures and the expression of the MyoD and MYOG antibodies. The study found that ADP MSCs show great clinical promise for muscle regeneration.

## 1. Introduction

Mesenchymal stem/stromal cells (MSCs), known as multipotent cells, hold great promise as a valuable resource in regenerative medicine due to their diverse differentiation capabilities, such as adipogenic, osteogenic, chondrogenic, and myogenic lineages [1,2]. MSCs also possess immunomodulatory properties [3]. The ability of MSCs to differentiate into muscle cells is one of their multipotency properties. It may play a crucial role in repairing and regenerating degenerated or lost muscle tissue.

Muscle degenerative diseases have no cure, and MSC transplantation offers one possibility for replacing damaged muscle. MSCs have several characteristics and biological properties that contribute to muscle regeneration. Their myogenic and angiogenic differentiation potentials help restore muscle mass, while their immunomodulatory properties mitigate muscle damage caused by chronic inflammation [4,5,6]. MSCs can enhance angiogenesis through direct differentiation or paracrine effects [6]. MSCs stimulate angiogenesis and tissue regeneration via paracrine effects, and the underlying mechanism appears to depend on fibroblast growth factor receptor (FGFR) and vascular endothelial growth factor receptor (VEGFR) signaling [5]. In mice, bone marrow-derived MSCs increased the expression of satellite cell-related genes and accelerated muscle protein turnover [7]; adipose-derived MSCs (ADP MSCs) differentiated into skeletal muscle satellite cells and ameliorated muscular dystrophy [8]; and amnion-derived MSC therapy improved muscle function in a Duchenne muscular dystrophy model [9]. MSC transplantation reduces fibroblast proliferation and prevents collagen accumulation through TGF-β3-dependent activation [10], which can aid in promoting the restoration of healthy muscle tissue. However, rat ADP MSCs are not commonly used in degenerated or lost muscle tissue. Furthermore, it is necessary to clarify the myogenic differentiation potential of MSCs and their mechanism for MSC clinical application in treating muscle disease. Injection of MSCs or MSC secretome has been shown to inhibit fatty degeneration and atrophy of rotator cuff muscles in sheep [11] and rats [12].

Understanding the immunomodulatory properties of MSCs is critical for developing therapeutic strategies that promote tissue regeneration. MSCs can enhance inflammation when the immune system is inactive and suppress inflammation when the immune system is overactive [13]. In other words, MSCs can potentially mitigate tissue damage and promote regenerative processes by suppressing excessive immune responses. Two different sources reported on the immunomodulatory properties of human MSCs. One study found that bone marrow-derived MSCs produce interleukin-6 (*IL-6*) [14]. The other found that placenta-derived MSCs secrete growth factors, express cytokines, such as *IL-6*, transform growth factor beta (*TGF-β*) [15], and are responsible for immunoregulation. MSCs produce growth factors such as fibroblast growth factor (*FGF*), hepatocyte growth factor (*HGF*), and insulin-like growth factor 1 (*IGF1*) and promote myoblast migration, multiplication, differentiation, and muscle regeneration [16,17,18].

However, the current knowledge of the myogenic differentiation and immunomodulatory properties of rat ADP MSCs is extremely limited, warranting further research to validate and expand our understanding. Therefore, this study evaluates the in-vitro myogenic differentiation capacity and immunomodulatory characteristics of ADP MSCs to ensure their suitability for clinical transplantation. This study hypothesized that rat ADP MSCs possess myogenic differentiation capabilities and exhibit immunomodulatory properties.

## 2. Materials and Methods

### 2.1. Animals and Study Design

**Figure 1:**
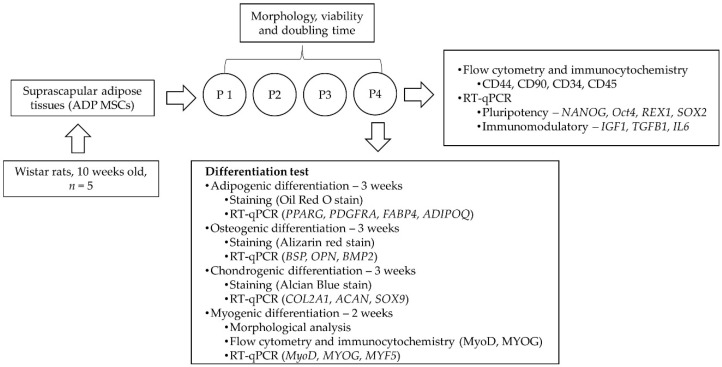
Experimental procedure. ADP MSCs; adipose-tissue-derived mesenchymal stem/stromal cells; P1–4, passages 1–4.

Five 10-week-old male Wistar rats were used to collect adipose tissues from which to isolate ADP MSCs. All animal experiments were performed in accordance with the approval guidelines of the Animal Care and Use Committee of the Tokyo University of Agriculture and Technology (approval no: R04-228). Rats were maintained in individual cages with a 12 h light/12 h dark cycle at a room temperature of 20.5 ± 2.5 °C. The experimental animals were fed and watered ad libidum. After harvesting adipose tissues for MSC isolation, the rats were kept alive for other studies. Figure 1 illustrates the overall structure of the experimental procedure.

### 2.2. Isolation and Culture of ADP MSCs

Cell isolation, culture, proliferation, and differentiation protocols followed those outlined in the previous studies [19,20,21,22]. ADP tissue collection was performed under anesthesia using the inhalation method: isoflurane 1–2% with an oxygen flow rate of 1–2 L/min. ADPs (3 g) were harvested from the subcutaneous fat in the periscapular region following an aseptic protocol.

The harvested ADPs were stored in a tube containing PBS (catalog no. 09-8912-100, Medicago AB, Uppsala, Sweden) and transported to the laboratory. Following PBS washing, the specimens were finely chopped in a 60 mm diameter culture dish (catalog no. TR4001, Nippon Genetics Co., Ltd., Tokyo, Japan) using sterile scissors within a biosafety cabinet. Next, the chopped ADPs were gently agitated in a shaking water bath for 1 h at 37 °C. The bath contained Hank’s balanced salt solution (product code 14025-092, Thermo Fisher Scientific Inc., New York, NY, USA) containing 0.1% collagenase type 1 (product code SCR103, Sigma-Aldrich, St. Louis, MO, USA). The collagenase activity was neutralized by adding DMEM (catalog no. 043-30085, FUJIFILM Wako Pure Chemical Corporation, Osaka, Japan) containing 20% fetal bovine serum (FBS) (catalog no. CCP-FBS-BR-500, COSMO BIO, Tokyo, Japan). Large aggregates were filtered out using a 100 µm filter (Minisart^®^, catalog no. S7597FXOSK, Sartorius Stedim Biotech, Göttingen, Germany). Subsequently, the cells were subjected to centrifugation at 800× *g* for 10 min, the supernatant was removed, and the cell pellets were resuspended and subjected to red blood cell (RBC) lysis using 1 mL of RBC lysis buffer (catalog no. 60-00051-10, pluriSelect Life Science UG (haftungsbeschränkt) & Co. KG, Leipzig, Germany). After a 10-min incubation at 4 °C with the RBC lysis buffer, the cells were washed with 10 mL of PBS. After centrifuging at 600× *g* for 3 min, the supernatant was discarded, and the cell pellets were resuspended in DMEM containing 20% FBS, 1% non-essential amino acids (catalog no. 139-15651, FUJIFILM Wako Pure Chemical Corporation, Osaka, Japan), and 1% Penicillin/Streptomycin (catalog no. 161-23181, FUJIFILM Wako Pure Chemical Corporation, Osaka, Japan) as a basal culture medium.

Culturing MSCs involves providing suitable conditions, including a specific growth medium, temperature, humidity, and gas pressure, to support MSC survival, proliferation, and differentiation. Cells were cultured in a 100 mm diameter culture dish (catalog no. TR4002, Nippon Genetics Co., Ltd., Tokyo, Japan) and kept at 37 °C in a humidified environment with 5% CO_2_. When cell confluency reached 80%, sub-culturing to the following passage was performed until passage 4.

### 2.3. Assessment of ADP MSCs’ Morphology, Viability, and Proliferation

Cell proliferation rates were evaluated based on the population doubling time. Cellular morphology and viability were assessed using an inverted microscope (Olympus CKX31, Tokyo, Japan) at ×40 magnification. Morphologically, MSCs were defined as spindle-shaped fibroblast-like cells. The average of cell viability and doubling time through passages 1–4 was performed by counting the cells at each passage using a Hirschmann counting chamber THOMA (product code 8100105, Hirschmann Laborgeräte GmbH & Co. KG, Eberstadt, Germany). Live cell percentages were calculated using the trypan blue exclusion method (product code 204-21102, FUJIFILM Wako Pure Chemical Corporation, Osaka, Japan). Both viability and doubling time served as indicators of proliferative performance. The doubling time was computed based on the time elapsed between passages (in days) and the number of living cells, employing the formula described below. Cell doubling time and viability data are presented using medians, with ranges obtained from five rats over four passages.

$$Doubling\;time=\frac{Duration\times log\left(2\right)}{log\left(final\;concentration\right)-log\left(initial\;concentration\right)}$$

### 2.4. Flow Cytometry and Immunocytochemistry Analysis of ADP MSCs

In passage 4, the ADP MSCs were analyzed using flow cytometry and immunocytochemistry to detect the standard MSC surface markers and hematopoietic cell markers. CD44 (catalog no. BS-0521R-FITC, Bioss, Woburn, MA, USA) and CD90 (PE anti-rat CD90, catalog no. 202523, BioLegend, San Diego, CA, USA) were employed as MSC surface marker antibodies. CD34 (catalog no. bs-0646R-FITC, Bioss, Woburn, MA, USA) and CD45 (PE anti-rat CD45, catalog no. 202207, BioLegend, San Diego, CA, USA) were employed as hematopoietic cell marker antibodies. Rabbit IgG isotype control (catalog no. bs-0295p-fitc, Bioss, Woburn, MA, USA) and PE Mouse IgG1, *κ* isotype control (catalog no. 400111, BioLegend, San Diego, CA, USA) were employed for isotype control.

For flow cytometry analysis, the cells underwent triple washes with PBS, and their concentrations were adjusted to 1 × 10^6^ cells/mL. These cell suspensions were then incubated in darkness for 20 min at 4 °C with the respective antibodies, following the manufacturer’s recommended concentrations. Afterward, any unbound antibodies were removed by rinsing with PBS. The flow cytometer (Beckman Coulter, Brea, CA, USA) was employed to detect cell surface antigens and assess their expression percentages, with data interpretation conducted via CytExpert Software version 2.3 (Beckman Coulter, Brea, CA, USA).

For immunocytochemistry analysis, cells were quantified and plated at a concentration of 2 × 10^4^ per well in a 24-well plate (catalog no. TR5002, Nippon Genetics Co., Ltd., Tokyo, Japan). After the confluency reached 80%, the cells were fixed by immersing them in 4% paraformaldehyde (catalog no. 09154-85, NACALAI TESQUE Inc., Kyoto, Japan) for 20 min at room temperature. Afterward, the cells were rinsed three times with a wash buffer containing 0.1% BSA (catalog no. A9418-5G, Sigma-Aldrich, St. Louis, MO, USA). The cells were then permeabilized in 0.1% Triton X-100 (catalog no. 9036-19-5, Sigma-Aldrich, St. Louis, MO, USA) in PBS for 10 min at room temperature. Next, the unspecific binding site was blocked by incubating the cells in 1% BSA in PBS for 1 h at room temperature. The primary antibodies were prepared by diluting them with 1% BSA, per the manufacturer’s recommended dilution rate. The antibodies were then incubated at room temperature for 2–3 h in the dark. Nuclear labeling was conducted using DAPI (product code 1351303, Bio-Rad Laboratories, Inc., Hercules, CA, USA) for 15 min at room temperature in the dark. After rinsing with wash buffer, the cells were visualized and fluorescently stained using an all-in-one fluorescence microscope equipped with the appropriate filters (BZ-9000, KEYENCE, Osaka, Japan).

### 2.5. Gene Expression of ADP MSCs by Reverse Transcription-Quantitative Polymerase Chain Reaction (RT-qPCR)

The procedure commenced by extracting the total RNA using the FastGene RNA Basic Kit (catalog no. FG-80250, Nippon Genetics Co., Ltd., Tokyo, Japan) following the manufacturer’s guidelines. To eliminate DNA contamination, the isolated RNA underwent treatment with the TURBO DNAfree™ Kit (catalog no. AM1907, Thermo Fisher Scientific Inc., New York, NY, USA). RNA quantity was assessed using a NanoDrop^TM^ Lite spectrophotometer (catalog no. ND-LITE-PR, Thermo Fisher Scientific Inc., Wilmington, DE, USA). Subsequently, the first-strand cDNA was synthesized using the ReverTra Ace^®^ qPCR RT Master Mix (code no. FSQ-201, TOYOBO, Osaka, Japan), according to the manufacturer’s instructions.

The expression levels were relatively quantified by utilizing the Applied Biosystems StepOnePlus™ Real-Time PCR System (Thermo Fisher Scientific, Waltham, MA, USA). The RT-qPCR setup included 2 µL cDNA, 0.5 µL of each forward and reverse primer (10 µmol/L), 10 µL THUNDERBIRD^®^ Next SYBR^®^ qPCR Mix (code no. QPX-201, TOYOBO, Osaka, Japan), and 7 µL dH_2_O. The thermal cycling conditions for qPCR were 30 s at 95 °C, followed by 40 amplification cycles, each comprising a denaturation step for 5 s at 95 °C and an annealing/extension step for 10 s at 60 °C. The relative quantification of gene expression was determined using the 2^−△△CT^ method and normalized to *beta-actin* as a reference gene.

The gene expression levels of pluripotent markers, including embryonic stem cell-specific homeobox (*NANOG*), octamer-binding transcription factor 4 (*Oct4*), reduced expression 1 (*REX1*), and SRY-box containing gene 2 (*SOX2*), were validated at the mRNA level through RT-qPCR. Immunomodulatory markers like transforming growth factor beta 1 (*TGFB1*), insulin-like growth factor 1 (*IGF1*), and interleukin 6 (*IL6*) were also validated in the same way. The relative expression level of *beta-actin* was presented as the median with a range (*n* = 5). Table 1 details the specific primers employed for the PCR reactions.

### 2.6. Trilineage and Myogenic Differentiation Potential of ADP MSCs

The plastic-adherent ADP MSCs in passage 4 were induced by the specific induction differentiation medium and were then evaluated for adipogenic, osteogenic, chondrogenic, and myogenic differentiation capacity. Adipogenic, osteogenic, and chondrogenic differentiation were verified by tissue-specific staining. Myogenic differentiation was validated based on morphological alterations, flow cytometry, and immunocytochemistry. Additionally, RT-qPCR was employed to analyze lineage-specific gene expression in the four lines. The standard culture medium served as a negative control for the undifferentiated ADP MSCs. For gene expression analysis, the relative expression level of *beta-actin* was presented as the median with a range (*n* = 5). Table 1 details the specific primers employed for the RT-qPCR reactions.

#### 2.6.1. Induction of ADP MSCs into Adipocyte

In a 6-well plate (catalog no. TR5000, Nippon Genetics Co., Ltd., Tokyo, Japan), cells were quantified and inoculated at a concentration of 1 × 10^5^ per well. At 80% confluence, an adipogenic-induction medium was introduced. This medium consisted of DMEM enriched with 20% FBS, 1 µM dexamethasone, 500 µM isobutylmethylxanthine (catalog no. AG-CR1-3512-G001, Adipogen Life Science Inc., San Diego, CA, USA), 100 µM indomethacin (catalog no. 405268, Sigma-Aldrich), and 5 µg/mL insulin (catalog no. 16634, Sigma-Aldrich, St. Louis, MO, USA). The medium was refreshed at three-day intervals for twenty-one days.

After 21 days of adipogenic induction in the induction medium, Oil Red O staining was conducted. Firstly, the cells were rinsed with PBS, fixed with 4% paraformaldehyde for 15 min, and stained with 0.5% Oil Red O (catalog no. O-0625, Sigma-Aldrich, St. Louis, MO, USA) in isopropanol-distilled water (3:2) for 10 min. This staining procedure was conducted to identify the presence of fat vacuole formation within the cell. The detection of adipocyte formation was performed using an inverted microscope at ×100 magnification. Fat vacuoles exhibiting a red stain within the cells, as revealed by Oil Red O staining, confirmed the formation of adipocytes.

Expression of the adipocyte marker genes peroxisome proliferator-activated receptor gamma (*PPARG*), platelet-derived growth factor receptor alpha (*PDGFR*), fatty acid binding protein 4 (*FABP4*), and adiponectin (*ADIPOQ*) was analyzed by RT-qPCR.

#### 2.6.2. Induction of ADP MSCs into Osteocyte

In a 6-well plate, cells were quantified and plated at a concentration of 1 × 10^5^ per well. Upon reaching 80% confluence, the osteogenesis differentiation medium was introduced. This medium composed of DMEM was combined with 20% FBS, 100 nM dexamethasone, 0.2 mM ascorbic acid (catalog no. 016-04805, FUJIFILM Wako Pure Chemical Corporation, Osaka, Japan), and 10 mM b-glycerol phosphate (catalog no. 17130-22, NACALAI TESQUE Inc., Kyoto, Japan). The medium was refreshed at three-day intervals for twenty-one days.

After 21 days of osteogenic induction in the induction medium, Alizarin Red (ALZ) staining was conducted. The cells underwent two PBS washes, followed by fixation in ice-chilled 70% ethanol for 1 h at 4 °C, and were then washed twice with dH_2_O. ALZ solution (catalog no. 40-1009-5, Sigma-Aldrich, St. Louis, MO, USA) was added to coat the cells thoroughly, followed by immersion for 30 min at room temperature. After conducting four washes of the wells with dH_2_O, images were captured using an inverted microscope at ×40 magnification. The red staining observed in the matrix mineral deposition confirmed the occurrence of osteogenic differentiation.

Expressions of the osteocyte marker genes, bone sialoprotein (*BSP*), osteopontin (*OPN*), and bone morphogenetic protein-2 (*BMP2*) were analyzed by RT-qPCR.

#### 2.6.3. Induction of ADP MSCs into Chondrocyte

Cells (1 × 10^5^) were plated in a 6-well plate and incubated at 37 °C in a humidified atmosphere with 5% CO_2_. The chondrogenic differentiation medium commenced once cell confluence reached 80%. The inductor medium consisted of DMEM, 20% FBS, 100 nM dexamethasone, 50 µg/mL ascorbic acid, and 10 ng/mL transforming growth factor (TGF) β3 (catalog no. HZ1090, Proteintech Group, Inc., Rosemont, IL, USA) and 1% premix ITS (catalog no. 354352, Corning Inc., New York, NY, USA), enriched with insulin, human transferrin, sodium selenite, and 40 µg/mL L-proline (catalog no. P0481, Tokyo Chemical Industry Co., Ltd., Tokyo, Japan). The medium was renewed at three-day intervals for 21 days.

After 21 days of chondrogenic induction in the induction medium, Alcian Blue staining was conducted. Cells were rinsed twice with PBS and fixed with 4% paraformaldehyde for 30 min at room temperature. After washing the cells with dH_2_O, 1% Alcian Blue (catalog no. 66011-100ML F, Sigma-Aldrich, St. Louis, MO, USA) was added and left at room temperature for the entire night while avoiding exposure to light. The next day, the staining reagent was removed, and the cells were rinsed 2–3 times with 0.1 N hydrochloric acid (HCL) (product code 083-01115, FUJIFILM Wako Pure Chemical Corporation, Osaka, Japan). After removing the HCL, cells were observed under an inverted microscope at ×40 magnification in the presence of dH_2_O. Alcian Blue staining allowed an evaluation of chondrocyte formation by highlighting highly sulfated proteoglycans within the cartilage matrix.

Expressions of the chondrocyte marker genes, collagen type II alpha 1 chain (*COL2A1*), aggrecan (*ACAN*), and SRY-box transcription factor 9 (*SOX9*) were analyzed by RT-qPCR.

#### 2.6.4. Induction of ADP MSCs into Myocyte

To determine the myogenic differentiation potential of ADP MSCs, cells were exposed to the myogenesis differentiation medium composed of DMEM/ F12 Medium (catalog no. 11320033, Life Technologies Corporation, Carlsbad, CA, USA), supplemented with 5% horse serum (catalog no. 16050130, Life Technologies Corporation, Carlsbad, CA, USA), 1% L-glutamine (catalog no. 074-00522, FUJIFILM Wako Pure Chemical Corporation, Osaka, Japan), 1 ng/mL FGF (catalog no. 10018B, PeproTech, Inc., Cranbury, NJ, USA), 10 ng/mL HGF (catalog no. 100-39H, PeproTech, Inc., Cranbury, NJ, USA), 10 ng/mL IGF (catalog no. 100-11, PeproTech, Inc., Cranbury, NJ, USA), and 0.4 μg/mL dexamethasone (catalog no. D4902, Sigma-Aldrich, St. Louis, MO, USA) [21,22]. Morphologically, myogenic cells were defined as elongated, aligned cells. The medium was renewed at three-day interval for twenty-one days.

#### 2.6.5. Analysis of the Myogenic Differentiated ADP MSCs

The cellular morphological alterations were monitored throughout the 14 days of myogenic differentiation using an inverted microscope at a magnification of ×40. Flow cytometry and immunocytochemistry were performed 14 days after induction of differentiation to detect myogenic-specific immunophenotypic markers, MyoD (catalog no. bs-2442R-FITC, Bioss, Woburn, MA, USA) and MYOG (catalog no. NBP2-33056PE, Novus Biologicals, Centennial, CO, USA). Rabbit IgG isotype control (catalog no. bs-0295p-fitc, Bioss, Woburn, MA, USA) and PE Mouse IgG1, *κ* isotype control (catalog no. 400111, BioLegend, San Diego, CA, USA) were used for isotype control.

For flow cytometry, the cells underwent triple washes with PBS, and their concentrations were adjusted to 1 × 10^6^ cells/mL. Cells were subjected to intracellular staining by passing through the fixation and permeabilization steps. For this purpose, the cells were fixed with 4% paraformaldehyde (catalog no. 09154-85, NACALAI TESQUE Inc., Kyoto, Japan) for 20 min at room temperature. Afterward, the cells were rinsed three times with wash buffer containing 0.1% BSA (catalog no. A9418-5G, Sigma-Aldrich, St. Louis, MO, USA). Then, the cells were permeabilized in 0.1% Triton X-100 (catalog no. 9036-19-5, Sigma-Aldrich, St. Louis, MO, USA) in PBS for 10 min at room temperature. Next, the unspecific binding site was blocked by incubating the cells in 1% BSA in PBS for 1 h at room temperature. The antibodies were prepared by diluting them with the blocking buffer per the manufacturer’s recommended dilution rate and incubating them with the respective antibodies in the dark for 20 min at 4 °C. Then, the cells were washed with wash buffer, 500 µL of 1% BSA in PBS was added, and they were subjected to the flow cytometer (Beckman Coulter, Brea, CA, USA) to detect the expression percentages. The data analysis used CytExpert Software version 2.3 (Beckman Coulter, Brea, CA, USA).

To conduct immunocytochemistry, cells were counted and seeded at a density of 2 × 10^4^ per well in a 24-well plate (catalog no. TR5002, Nippon Genetics Co., Ltd., Tokyo, Japan). Cell fixation, permeabilization, and antibody preparation were performed for intracellular staining using the same method as for flow cytometry. They were then incubated with the respective antibodies for 2–3 h at room temperature in the dark. Then, nuclear labeling with DAPI was performed (product code 1351303, Bio-Rad Laboratories, Inc., CA, USA) for 15 min at room temperature in the dark. After rinsing with wash buffer, the cells were strained using an all-in-one fluorescence microscope equipped with the appropriate filters (BZ-9000, KEYENCE, Osaka, Japan).

Expression of two myogenic-related genes, myogenic differentiation 1 (*MyoD*), myogenin (*MYOG*), and myogenic factor 5 (*MYF5*), was analyzed by RT-qPCR.

### 2.7. Evaluation and Interpretation of Statistical Data

The RT-qPCR results—encompassing data related to adipogenic, osteogenic, chondrogenic, and myogenic differentiation as compared to undifferentiated conditions—underwent statistical analysis utilizing GraphPad Prism software version 8.4 (GraphPad Software, Inc., La Jolla, CA, USA). Data not following a normal distribution are presented as the median with range, and between-group comparisons were performed using the Mann–Whitney test. The level of significance was set at *p* < 0.05.

## 3. Results

### 3.1. Cell Morphology, Viability, Proliferation, and Gene Expression in Rat ADP MSCs

**Figure 2:**
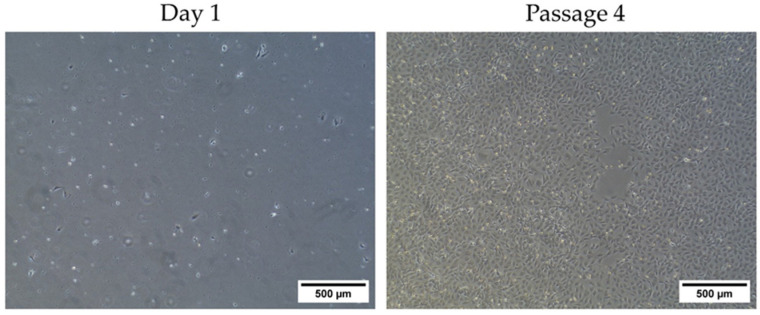
Cell morphology of plastic-adherent rat ADP MSC culture at Day 1 and Passage 4. The scale bar indicates a length of 500 µm.

After 24 h, MSCs began adhering to the plastic culture dish. In subsequent passages, spindle-shaped fibroblast-like cells exhibiting typical MSC morphology became evident (Figure 2). The median values (range) for the viability (%) and doubling time (days) of the ADP MSCs were 92 (90.5–92.8) and 1.9 (1.6–4.4), respectively.

### 3.2. Immunophenotypic Characterization and Gene Expression of ADP MSCs

**Figure 3:**
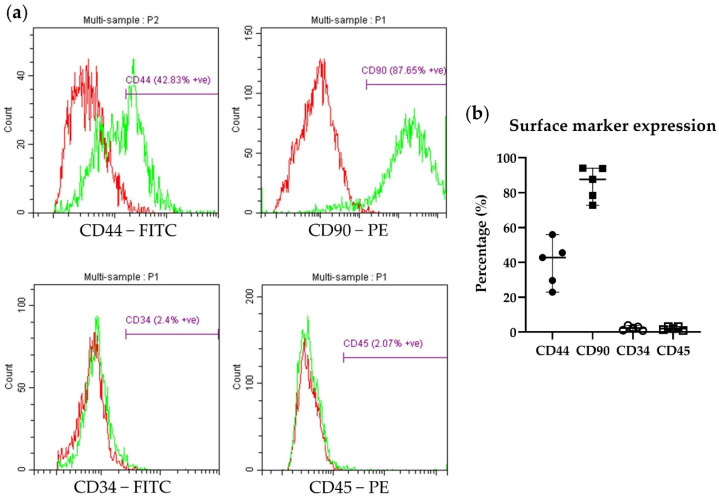
(**a**) Exemplary histograms of MSC surface marker expressions of CD44 and CD90 and hematopoietic marker expression of CD34 and CD45 in rat ADP MSCs. The red histogram represents isotype control, and the green histogram represents respective antibodies. (**b**) Flow cytometry analysis of the cell surface marker expression in passage 4 of rat ADP MSCs. Data are presented as the median (wide horizontal line) with range (narrow horizontal line) of the positive expression percentage of MSC markers (CD44; filled circle and CD90; filled rectangle) and hematopoietic cell markers (CD34; unfilled circle and CD45; unfilled rectangle) (*n* = 5). CD: cluster of differentiation; FITC: fluorescein isothiocyanate; PE: phycoerythrin.

The exemplary histograms and percentage of cell surface marker expression detected by flow cytometry are presented as the median (range) (Figure 3). The median (range) expression of the MSCs-specific markers CD44 and CD90 was 42.8% (23.0–56.0) and 87.7% (72.8–94.0) (*n* = 5), respectively. The median (range) expression of the hematopoietic cell markers CD34 and CD45 was 2.4% (0.9–3.9) and 2.1% (0.9–3.2) (*n* = 5), respectively.

**Figure 4:**
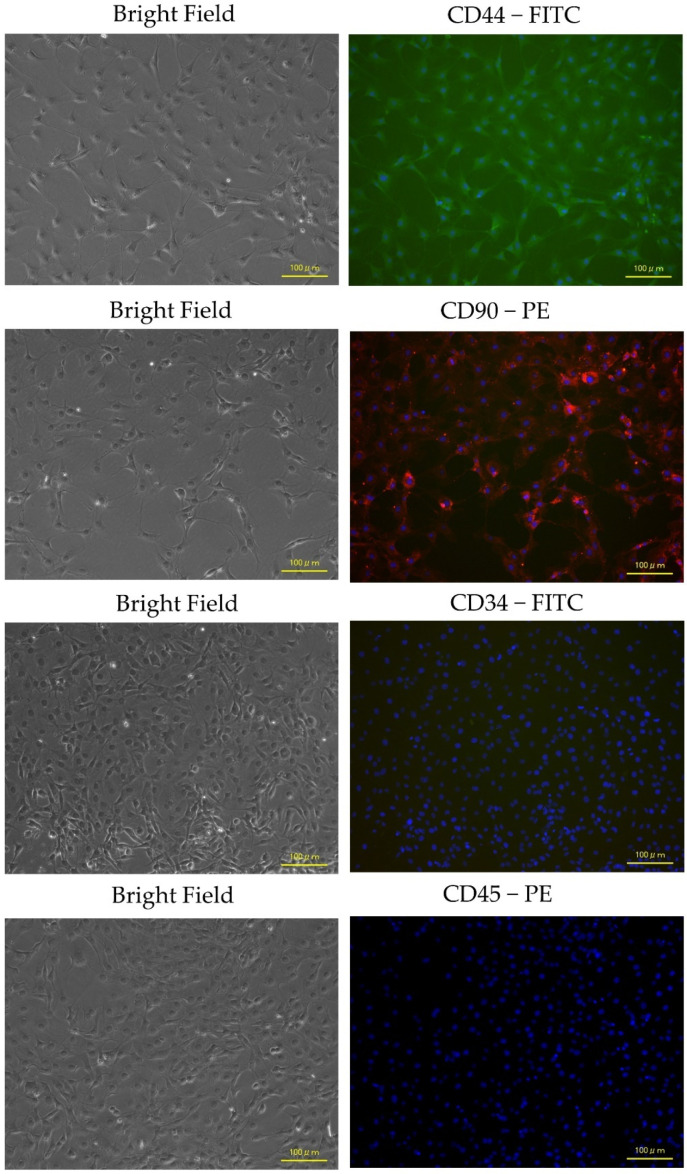
Representative immunocytochemistry images confirmed the expression of the MSCs-specific surface markers CD44 and CD90, with no expression of hematopoietic markers CD34 and CD45 in rat ADP MSCs. Nuclei were visualized by staining with blue fluorescence DAPI. The scale bar indicates a length of 100 µm. DAPI; 4′,6-diamidino-2-phenylindole.

Figure 4 presents representative immunocytochemistry images. Immunocytochemistry confirmed positive expression of MSC-specific surface markers (CD44 and CD90) and no expression of hematopoietic markers (CD34 or CD45).

**Figure 5:**
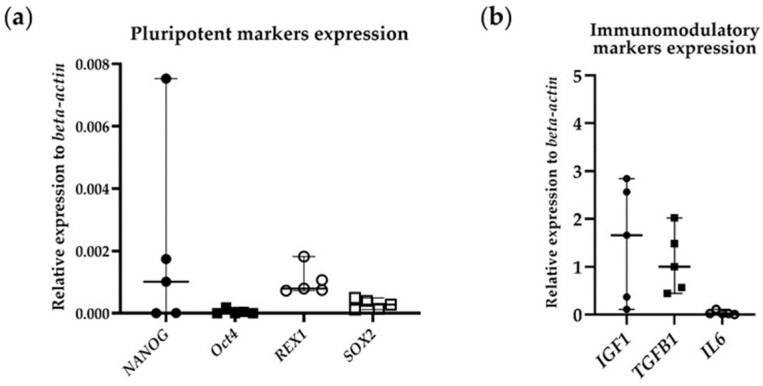
(**a**) RT-qPCR analysis of pluripotent markers (*NANOG*; filled circle, *Oct4*; filled rectangle, *REX1*; unfilled circle, *SOX2*; unfilled rectangle) and (**b**) immunomodulatory markers (*IGF1*; filled circle, *TGFB1*; filled rectangle, *IL6*; unfilled circle) expression in passage 4 of rat ADP MSCs. Data are shown as the relative expression to *beta-actin* and presented as the median (wide horizontal line) with range (narrow horizontal line) (*n* = 5).

Figure 5 shows the gene expression of the pluripotent and immunomodulatory markers. ADP MSCs expressed the pluripotent marker genes *NANOG* and *REX1* at a high level, while *Oct4* and *SOX2* were expressed at a low level. They also expressed the immunomodulatory marker genes IGF1 and TGFB1 at high levels but IL6 at low levels.

### 3.3. Differentiation Potential of Rat ADP MSCs

#### 3.3.1. Adipogenic Potential

**Figure 6:**
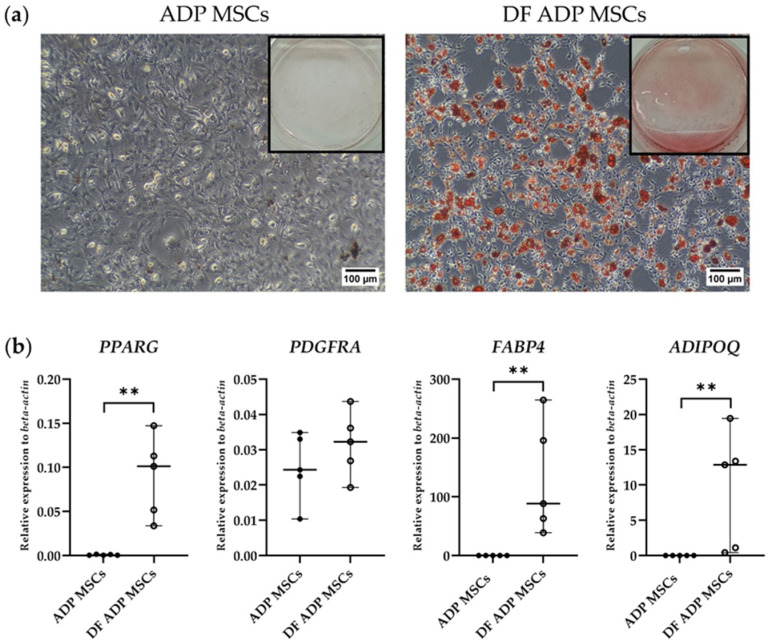
(**a**) Representative histological and 6-well images of Oil Red O staining of the adipogenic differentiated and undifferentiated rat ADP MSCs. The scale bar indicates a length of 100 µm. (**b**) RT-qPCR analysis of adipogenic cell markers (*PPARG*, *PDGFRA*, *FABP4*, and *ADIPOQ*) gene expression. Data on the relative expression of *beta-actin* were analyzed with the Mann–Whitney test and presented as the median (wide horizontal line) with range (narrow horizontal line) (*n* = 5) (** *p* < 0.01). ADP MSCs (filled circle); DF ADP MSCs, differentiated ADP MSCs (unfilled circle).

Adipogenic differentiated ADP MSCs showed distinct fat vacuole formation by Oil Red O staining, whereas no observable red staining of lipid droplets was observed before the cultures were differentiated (Figure 6a).

Gene expression analysis showed that differentiated ADP MSCs (DF ADP MSCs) highly expressed the adipogenic-related genes (*PPARG*, *PDGFRA*, *FABP4*, and *ADIPOQ*) (Figure 6b). The relative expression levels of *PPARG*, *FABP4*, and *ADIPOQ* in the DF ADP MSCs were significantly elevated compared to the before-differentiated MSCs (ADP MSCs) (*p* = 0.008).

#### 3.3.2. Osteogenic Potential

**Figure 7:**
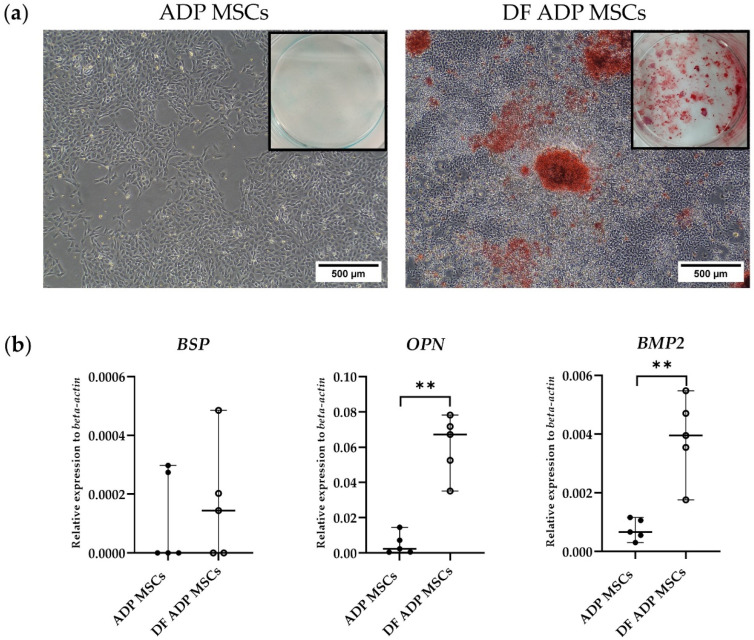
(**a**) Representative histological and 6-well images of Alizarin Red staining of the osteogenic differentiated and undifferentiated rat ADP MSCs. The scale bar indicates a length of 500 µm. (**b**) RT-qPCR analysis of osteogenic cell markers (*BSP*, *OPN*, and *BMP2*) gene expression. Data on the relative expression of *beta-actin* were analyzed with the Mann–Whitney test and presented as the median (wide horizontal line) with range (narrow horizontal line) (*n* = 5) (** *p* < 0.01). ADP MSCs (filled circle); DF ADP MSCs (unfilled circle).

ALZ-positive staining confirmed the occurrence of osteogenesis by staining the matrix minerals secreted from differentiated ADP MSCs. In contrast, the undifferentiated control exhibited no red-colored ALZ staining (Figure 7a).

In the gene expression analysis, the DF ADP MSCs highly expressed the osteogenic-related genes (*BSP*, *OPN*, and *BMP2*) (Figure 7b). The relative expression levels of *OPN* and *BMP2* in the DF ADP MSCs were significantly upregulated compared to the ADP MSCs (*p* = 0.008). While the expression of the *BSP* gene did not display statistically significant differences, it did exhibit an upward trend in the differentiated cells.

#### 3.3.3. Chondrogenic Potential

**Figure 8:**
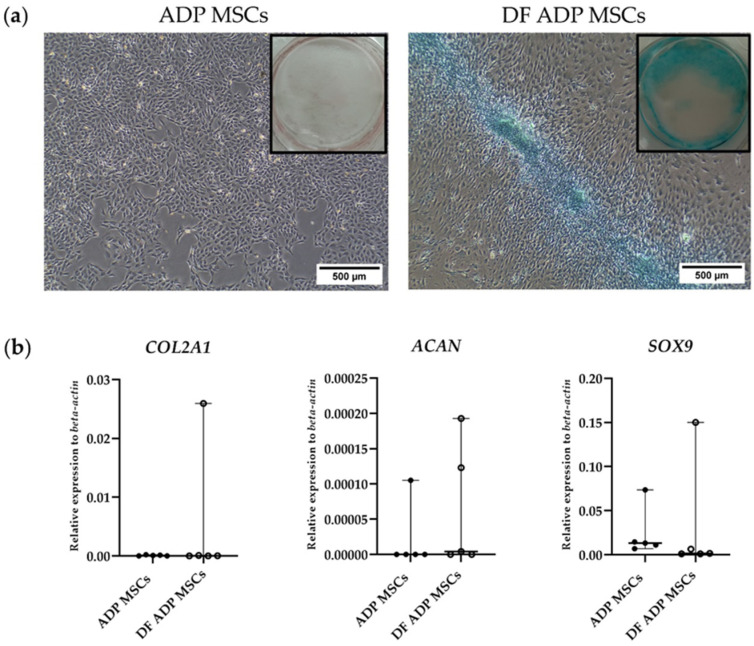
(**a**) Representative histological and 6-well images of Alcian Blue staining of the chondrogenic differentiated and undifferentiated rat ADP MSCs. The scale bar indicates a length of 500 µm. (**b**) RT-qPCR analysis of chondrogenic cell markers (*COL2A1*, *ACAN*, and *SOX9*) gene expression. Data on the relative expression of *beta-actin* were analyzed with the Mann–Whitney test and presented as the median (wide horizontal line) with range (narrow horizontal line) (*n* = 5). ADP MSCs (filled circle); DF ADP MSCs (unfilled circle).

Alcian Blue staining revealed chondrogenesis by positive staining of cartilage matrix components in DF ADP MSCs. In contrast, the undifferentiated control exhibited no blue staining (Figure 8a).

In the gene expression analysis, the DF ADP MSCs expressed the chondrogenic-related genes (*COL2A1*, *ACAN*, and *SOX9*). However, the expression levels were not statistically significant compared to the ADP MSCs (Figure 8b).

#### 3.3.4. Myogenic Potential

**Figure 9:**
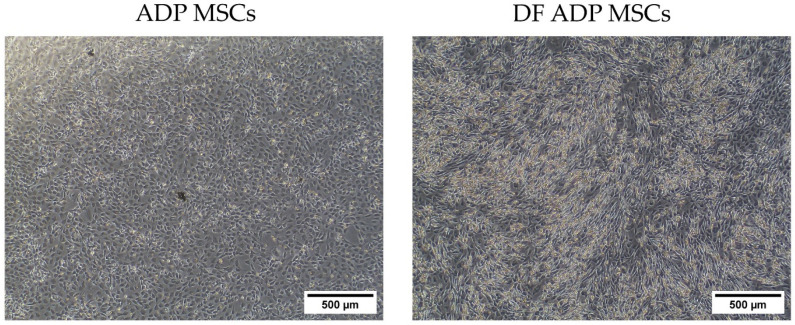
Morphological changes of the myogenic differentiated and undifferentiated rat ADP MSCs. The scale bar indicates a length of 500 µm.

From 3 to 14 days after the induction of myogenic differentiation, the morphology of MSCs changed to elongated, aligned, myocyte-like DF ADP MSCs (Figure 9).

**Figure 10:**
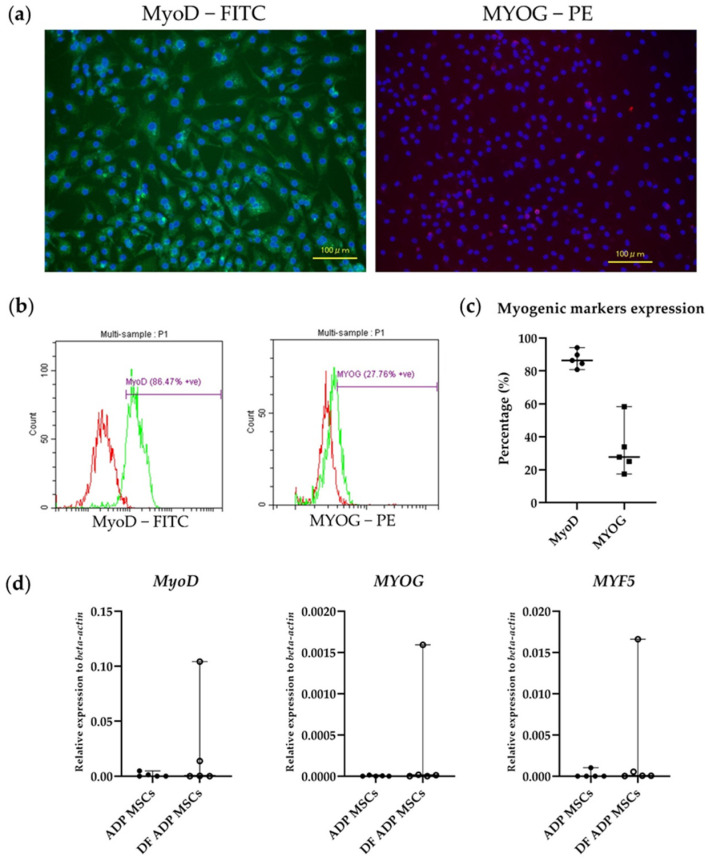
(**a**) Illustrative immunocytochemistry images, (**b**) flow cytometry histograms, and (**c**) flow cytometry analysis confirmed the expression of myogenic-specific markers (MyoD and MYOG) in myogenic differentiated rat ADP MSCs. (**a**) Nuclei were visualized by staining with blue fluorescence DAPI. The scale bar indicates a length of 100 µm. (**b**) The red histogram represents isotype control, and the green histogram represents respective antibodies. (**c**) Data are presented as the median (wide horizontal line) with range (narrow horizontal line) of the positive expression percentage of myogenic cell markers (MyoD; filled circle and MYOG; filled rectangle) (*n* = 5). (**d**) RT-qPCR analysis of myogenic cell markers (*MyoD*, *MYOG*, and *MYF5*) gene expression. Data on the relative expression of *beta-actin* were analyzed with the Mann–Whitney test and presented as the median (wide horizontal line) with range (narrow horizontal line) (*n* = 5). ADP MSCs (filled circle); DF ADP MSCs (unfilled circle).

Immunocytochemistry confirmed the FITC expression of MyoD, and PE expression of MYOG was localized around the nucleus (Figure 10a). In the flow cytometry results, the median (range) expression percentage of the myogenic markers MyoD and MYOG was 86.5% (80.9–94.2) and 27.8 (17.5–58.4), respectively (*n* = 5) (Figure 10b,c).

The relative expression levels of the myogenic-related genes (*MyoD*, *MYOG*, and *MYF5*) in the DF ADP MSCs were elevated only in a few samples (Figure 10d).

## 4. Discussion

In ADP MSCs collected from rats’ periscapular regions, MSC surface markers CD44 and CD90 were detected, as well as the differentiation potential into three lineages, expression of the pluripotent marker genes *NANOG* and *REX1*, and the immunomodulatory marker genes *IGF1* and *TGFB1*. Rat ADP MSCs also showed differential potential in myogenic cells. These findings suggest the possibility of using MSCs for muscle regeneration.

ADP MSCs are a promising alternative to bone marrow-derived MSCs. ADP MSCs are easier to isolate and proliferate more rapidly than bone marrow-derived MSCs, making them a promising candidate for use in musculoskeletal disease [23]. Human ADP MSCs had higher myogenic differentiation potential than bone marrow-derived MSCs and synovial MSCs [24]. The myogenic differentiation potential of bone marrow-derived MSCs and gonadal fat pad-derived MSCs in rats was similar [23]. In addition, the ADP MSCs’ characteristics have been shown to vary by anatomical sources [24]. The adipose tissues in the scapular region are brown adipose tissues, while white adipose tissue surrounds the internal organs [25,26]. Fat isolation around the periscapular region is not only more accessible than intra-abdominal fat harvesting but also has a lower risk of potential complications and minimal discomfort. For these reasons, we used rat ADP MSCs harvested from the periscapular region.

MSCs are evaluated based on established criteria such as morphological characteristics, surface markers, and multipotent differentiation abilities. First, the rat ADP MSCs used in this study were plastic-adherent and exhibited a spindle-shaped, fibroblast-like morphology typical of MSCs [20,27]. The median (range) viability (%) of cultured cells in passages 1–4 was 92 (90.5–92.8), with a doubling time (days) of 1.9 (1.6–4.4). The previously reported doubling time of rat ADP MSCs derived from inguinal fat was an average of 39.9 ± 4.4 h (1.6 days) [28]. To the best of our knowledge, there are no reports on the viability of rat ADP MSCs. Cellular viability and proliferative potential are important for MSCs’ effective functional performance.

Second, the surface markers of rat ADP MSCs, CD44 and CD90, were highly detected by flow cytometry and immunocytochemistry; hematopoietic cell markers, CD34 and CD45, were minimally expressed [23,29,30]. However, the expression rate of CD44 by flow cytometry was 42.8%. The expression rate of surface markers in MSCs varies with tissue origin. Hendawy et al. [31] reported a high variation in CD44 expression in rat ADP stromal cells isolated from the falciform ligament and periovarian region, ranging from 17.94% to 45.25%. In native and fresh mouse bone marrow-derived MSCs, CD44 expression is 45%, but CD44 is not expressed in most human bone marrow-derived MSCs [32]. These MSCs will express CD44 in culture. In this study, although CD44 expression was relatively low, these cells were considered MSCs based on their morphological characteristics, other surface markers, and ability to differentiate into adipocytes, osteocytes, chondrocytes, and myogenic cells.

The third criterion for MSCs is multipotency. Rat ADP MSCs showed differentiation capacity into adipocytes, osteocytes, chondrocytes, and myogenic cells. Transcription factors known for their role in preserving pluripotency in embryonic stem cells, such as *NANOG*, *Oct4*, *REX1*, and *SOX2* [33], have been proposed to play similar roles in adult stem cells. Identifying pluripotent markers in MSCs is crucial for assessing their regenerative potential and stem cell characteristics [34]. In this study, we found high expressions of *NANOG* and *REX1* but low expressions of *Oct4* and *SOX2*. These results closely align with previous research findings [35], which confirmed the expression of *NANOG* in human MSCs and found no expression of *Oct4* and *SOX2*, but found that *NANOG* expression may vary with the passaging of the cells. Hendway et al. [19] reported that *REX1* was expressed in ADP MSCs, but *NANOG*, *Oct4*, and *SOX2* were not expressed. Therefore, we concluded that cell passaging may alter *NANOG* expression, whereas *Oct4* and *SOX2* are either not expressed or expressed at lower levels in adult MSCs.

Genes associated with adipogenesis, such as *PPARG* [36], *FABP4* [37], and *ADIPOQ* [38], were detected in the adipogenesis of rat ADP MSCs. *PDGFRA* expression was not significantly different compared to the undifferentiated MSCs. *PDGFRA* is platelet-derived growth factor receptor alpha. According to Uezumi et al. [39], *PDGFRA*-positive cells efficiently differentiate into adipocytes. However, after adipogenesis, the cells were no longer *PDGFRA*-positive. It is inferred that *PDGFRA* functions as a commitment marker for adipogenic differentiation of MSCs; based on the results of this study, *PPARG*, *FABP4*, and *ADIPOQ* can be used as adipogenic markers 3 weeks after inducing differentiation.

Gene association osteogenesis, such as *OPN* [40,41] and *BMP2* [42], were detected in the bone differentiation of rat ADP MSCs. *BSP* expression was not significantly different compared to that of undifferentiated MSCs. *BSP* is an important contributor to bone mineralization, and its expression is associated with growth factors such as *IGF1* and *TGFB1* [43]. Additionally, the low expression of *BSP* in our study could be explained by the fact that *BSP* is an early-stage marker of osteogenic differentiation [44], and its expression may change throughout differentiation. Based on the results of this study, *BSP* can be used as a marker for early-stage osteogenic differentiation, while *OPN* and *BMP2* can be used as osteogenic markers 3 weeks after inducing differentiation.

Regarding gene associations with chondrogenesis, such as *COL2A1* [45], *ACAN*, and *SOX9*, no significant differences were observed between differentiated and undifferentiated MSCs. *COL2A1* is an early-stage chondrogenesis marker [46]. *ACAN* is a major proteoglycan and is the most abundant non-collagenous protein in articular cartilage [47]. *ACAN* is constitutively expressed in undifferentiated MSCs [46]. *SOX9* was the first transcription factor discovered in stem cells [48]. *SOX9* contributes to differentiation commitment [49] and is essential for chondrogenic differentiation [50]. The results of this study indicated that these genes cannot be used as markers of chondrogenic differentiation. We suggest using *COL2A1* as an early-stage marker for chondrogenic differentiation; caution should be taken when using *ACAN* and *SOX9* as chondrogenic markers.

The immunomodulatory properties of MSCs are regarded as potentially valuable priming factors to enhance the therapeutic efficacy of MSCs or to explore alternative therapeutic strategies. This study examined the gene expression of immunomodulatory markers and found that *IGF1* and *TGFB1* were expressed. *IGF1* is a growth factor involved in cell growth, proliferation, and tissue repair [51]. Human ADP MSCs express and secrete significant amounts of *IGF1* (328.3 +/− 22.7 pg/µg DNA) [52]. Rat ADP MSCs expressing *IGF1* secreted higher levels of *IGF1* and improved the prognosis of myocardial infarction [29]. *TGFB1* is a multifunctional cytokine and plays a crucial role in various cellular processes, including cell growth and differentiation, immunosuppression, and tissue repair [53]. *IL6* is a multifunctional cytokine that can have pro- and anti-inflammatory effects on the immune system [54]. Park et al. [14] reported that MSCs produce *IL6*, which is responsible for the immunoregulatory effects of MSCs. The low expression of *IL6* in this study may be due to the absence of infection or inflammation that causes *IL6* expression [55] in normal MSCs. Immunomodulatory properties of MSCs also include the suppression of T cell proliferation, influence on dendritic cell maturation and function, suppression of B cell proliferation and terminal differentiation, and modulation of other immune cells such as NK cells and macrophages [56].

Appropriate differentiation medium and culture methods are required to achieve myogenic differentiation of ADP MSCs. However, the most efficient differentiation conditions for differentiating ADP MSCs into functional muscle progenitor cells have not yet been established. This study induced ADP MSCs into myogenic cells using a culture medium supplemented with the growth factors FGF, HGF, and IGF [21,22]. FGFs and HGFs regulate satellite cell proliferation. IGF plays a major role in regulating cell growth, stimulating myoblast differentiation, and enhancing protein synthesis in differentiated myofibers [16,17,57]. The myogenic differentiation of rat ADP MSCs was confirmed by morphological changes that transformed them into elongated and aligned structures.

Flow cytometry, immunocytochemistry, and RT-qPCR detected myogenic markers in the differentiated cells. Flow cytometry focuses on antigen expression, while RT-qPCR focuses on genetic material; these two methods cannot simply substitute each other at a single point in time [58]. *MyoD*, *MYOG*, and *MYF5* are myogenic regulators. They are recognized as members of the basic helix-loop-helix family of transcription factors that control the determination and differentiation of skeletal muscle cells during embryogenesis and postnatal myogenesis [59,60]. No significant differences in *MyoD*, *MYOG*, and *MYF5* expressions were observed between differentiated and undifferentiated MSCs. Gang et al. [61] reported that *MyoD* and *MYOG* are early myogenic markers and are not expressed 2 weeks after inducing differentiation. Therefore, detecting them at an earlier stage of differentiation may be desirable, though myosin heavy chain (*MHC*) could serve as a late myogenic marker. The differentiation potential of MSCs has been shown to relate to the age of the animal supplied, the source of cell collection [62], the medium composition [22], and the number of passages of cultured cells [63]. It has also been reported that satellite cell markers in mice may not be used in humans [64]. Therefore, the expression of myogenic markers may also be affected by species-specific factors, tissue origin, cell passages, and the composition of the differentiation medium. Based on these results, we suggest confirming the myogenic differentiation potential of rat ADP MSCs by performing at least two of the following methods: flow cytometry, immunocytochemistry, and RT-PCR.

A limitation of this study is that the expression levels of myogenic genes varied among the samples, and the overall expression of these genes was low. The reasons for this need further investigation. The data variability is due not only to the number of samples but to many other factors. These include the time of evaluation during differentiation, the heterogeneity of ADP MSCs during myogenic differentiation, the composition of the differentiation medium, or the use of a single-time point analysis of differentiated cells. In the future, we would like to increase the number of samples and measure early and late differentiation processes to clarify the cause of the variation in the data. There are no reports on the immunomodulatory effects of MSCs on fatty muscle degeneration. The next step is to develop methods to evaluate the regenerative and immunomodulatory effects of MSCs in vivo to determine whether the differential potential of MSCs or the immunomodulatory effects of MSCs are responsible for the mechanism of muscle regeneration.

## 5. Conclusions

In rat ADP MSCs, CD44 and CD90 were detected as surface markers, along with the potential for differentiation into three lineages. The expression of the pluripotent marker genes *NANOG* and *REX1* was also confirmed. The expression of the immunomodulatory marker genes *IGF1* and *TGFB1* suggested that they have immunomodulatory properties. Furthermore, the capacity of rat ADP MSCs to differentiate into myogenic cells was confirmed by the morphology and expression of MyoD and MYOG antibodies. According to the results of this study, rat ADP MSCs hold great promise for clinical applications in muscle regeneration.

## Figures and Tables

**Table 1:** Nucleotide sequence of primers used in this study.

Gene Name		Direction	Primer Sequences (5′–3′)
Housekeeping gene	*beta-actin*	Forward	GCAGGAGTACGATGAGTCCG
		Reverse	ACGCAGCTCAGTAACAGTCC
Pluripotent marker genes	*NANOG*	Forward	TACCTCAGCCTCCAGCAGAT
		Reverse	CATTGGTTTTTCTGCCACCT
	*Oct4*	Forward	CGAACCTGGCTAAGCTTCCA
		Reverse	GCCATCCCTCCACAGAACTC
	*REX1*	Forward	GCTCCGGCGGAATCGAGTGG
		Reverse	GCACGTGTTGCTTGGCGACC
	*SOX2*	Forward	CTCGCAGACCTACATGAAC
		Reverse	TCGGACTTGACCACAGAG
Immunomodulatory marker genes	*IGF1*	Forward	TGGTGGACGCTCTTCAGTTC
		Reverse	TCCGGAAGCAACACTCATCC
	*TGFB1*	Forward	ATGCCAACTTCTGTCTGGGG
		Reverse	GGTTGTAGAGGGCAAGGACC
	*IL6*	Forward	CCACCCACAACAGACCAGTA
		Reverse	TCTGACAGTGCATCATCGCT
Adipogenic genes	*PPARG*	Forward	AGCTCTGTGGACCTCTCTGT
		Reverse	GTCAGCTCTTGTGAACGGGA
	*PDGFRA*	Forward	AGTGCTTGGTCGGATCTTGG
		Reverse	GAGCATCTTCACAGCCACCT
	*FABP4*	Forward	AACTGGGCGTGGAATTCGAT
		Reverse	CACATGTACCAGGACCCCAC
	*ADIPOQ*	Forward	TAATTCAGAGCAGCCCGTAG
		Reverse	TGGGGATAACACTCAGAACC
Osteogenic genes	*BSP*	Forward	AGGCTACGAGGGTCAGGATT
		Reverse	GCACCTTCCTGAGTTGAGCT
	*OPN*	Forward	GAAGAGCCAGGAGTCCGATG
		Reverse	CTTCCCGTTGCTGTCCTGAT
	*BMP2*	Forward	CAGGTCTTTGCACCAAGATG
		Reverse	GCTGGACTTAAGACGCTTCC
Chondrogenic genes	*COL2A1*	Forward	TCCTAAGGGTGCCAATGGTGA
		Reverse	AGGACCAACTTTGCCTTGAGGAC
	*ACAN*	Forward	CTCTGCCTCCCGTGAAAC
		Reverse	TGAAGTGCCTGCATCTATGT
	*SOX9*	Forward	GAAAGACCACCCCGATTACAAG
		Reverse	AAGATGGCGTTAGGAGAGATGTG
Myogenic genes	*MyoD*	Forward	CGACTCTTCAGGCTTGGGTT
		Reverse	TGTCGCAAAGGAGCAGAGAG
	*MYOG*	Forward	GGCAATGCACTGGAGTTTGG
		Reverse	CGTAAGGGAGTGCAGGTTGT
	*MYF5*	Forward	ATGGACATGACGGACAGCTG
		Reverse	TGCGACTCTTGGCTCAAACT

## Data Availability

Data are contained within the article.
